# Nutrition in the First 1000 Days: Ten Practices to Minimize Obesity Emerging from Published Science

**DOI:** 10.3390/ijerph14121491

**Published:** 2017-12-01

**Authors:** Angelo Pietrobelli, Massimo Agosti, the MeNu Group

**Affiliations:** 1 Pediatric Unit, Verona University Medical School, Piazzale A. Stefani, 37126 Verona, Italy; angelo.pietrobelli@univr.it; 2 Pennington Biomedical Research Center, Baton Rouge, LA 70808, USA; 3 Neonatology and Neonatal Intensive Unit Care, Maternal & Child Department del Ponte Hospital, Azienda Ospedaliera di Circolo Fondazione Macchi, 21100 Varese, Italy; massimo.agosti@ospedale.varese.it

**Keywords:** nutrition, prevention, pediatric obesity, growth, first 1000 days

## Abstract

The prevalence of childhood overweight and obesity has increased in most countries the last decades. Considering this in a simplistic way, we can say that obesity is the result of an imbalance between energy intake and energy expenditure. Moreover, the environment from conception to childhood could influence the child’s future health. The first 1000 days of life start with woman’s pregnancy, and offer a unique window of opportunity to contribute to obesity prevention. In light of the actual literature, the aim of our article is to discuss a proposal of 10 good practices to minimize obesity in the first 1000 days emerging from published science. (1) Both the mother’s and the father’s behaviors are important. A balanced diet with appropriate fat and protein intake, and favoring fruits and vegetables, is recommended for both parents during the conception period and pregnancy. Furthermore, overweight/obese women who are planning to become pregnant should reduce their weight before conception. (2) During pregnancy, at birth, and during early life, body composition measurements are crucial to monitor the baby’s growth. (3) Exclusive breastfeeding is recommended at the beginning of life until six months of age. (4) Four to six months of age is the optimal window to introduce complementary feeding. Until one year of age, breast milk or follow-on/commercial formula is the main recommended feeding source, and cow’s milk should be avoided until one year of age. (5) Fruit and vegetable introduction should begin early. Daily variety, diversity in a meal, and repeated exposure to the food, up to eight times, are efficient strategies to increase acceptance of food not well accepted at first. There is no need to add sugar, salt, or sugary fluids to the diet. (6) Respect the child’s appetite and avoid coercive “clean your plate” feeding practices. Adapt the portion of food and don’t use food as reward for good behavior. (7) Limit animal protein intake in early life to reduce the risk of an early adiposity rebound. Growing-up milk for children between one and three years of age should be preferred to cow’s milk, in order to limit intake and meet essential fatty acid and iron needs. (8) The intake of adequate fat containing essential fatty acids should be promoted. (9) Parents should be role models when feeding, with TV and other screens turned-off during meals. (10) Preventive interventions consisting of promoting physical activity and sufficient time dedicated to sleep should be employed. In fact, short sleep duration may be associated with increased risk of developing obesity. Based on literature reviews, and given the suggestions described in this manuscript, concerted public health efforts are needed to achieve the healthy objectives for obesity and nutrition, and to fight the childhood obesity epidemic.

## 1. Introduction

The incidence of pediatric obesity worldwide in 2010 was estimated at 43 million overweight and obese preschool children (i.e., >2 SDs above the median WHO standards) in developing and developed countries. In addition according with De Onis et al. [1], 92 million preschool children are estimated to be at risk of overweight (>1 SD and ≤2 SDs of the median weight-for-height).

The first 1000 days of life—the period from conception to age of two—is the most important period for the body and brain development, and it represents the best time for obesity prevention and its adverse consequences [2,3,4,5,6]. There are many growth drivers during this complex phase of life, among them nutrition, genetic and epigenetic factors, and hormonal regulation. The challenge thus involves maximizing the potential for normal growth without increasing the risk of associated disorders. Woo Baidal et al. very recently in a omni-comprehensive review article, while keeping in mind modifiable risk factors for childhood obesity occurring in the first 1000 days of life, indicated strong evidence for risk factors during pregnancy (i.e., high maternal pre-pregnancy body mass index, excess gestational weight gain, gestational diabetes, tobacco exposure), and infancy (i.e., high infant birth weight, accelerated infant weight gain), as well as other factors (i.e., parent-infant relationship, infant sleep, inappropriate bottle use, introduction of solid food before four months of life) [7]. Identification of effective early-life intervention targeting these modifiable factors is critical for pediatric as well as adult obesity prevention [8].

Using the 2011 Cochrane review of 55 intervention studies for preventing obesity in children from birth [9], and other review articles where intervention in children, feeding, and lifestyle practices were presented [10,11,12], we developed and discuss a proposal of ten good practices to help prevent obesity in the unique window of opportunity to contribute to obesity prevention in the first 1000 days of life. Clearly, based on literature review, we are proposing these ten practices to minimize obesity emerging from published science.

## 2. Practice One: Both Mother and Father Behavior Are Important

Julie A. Mennella, suggested recently that one approach to improving infant diet quality, and reducing child obesity risk, may be through influencing the diet behaviors of their parents [13]. Infant size related to both pre-pregnancy obesity and greater-than recommended gestational weight gain are independently associated with risk for more rapid post-natal growth [13], even in preterm infants [14]. Community health workers providing education on maternal diet in pregnant women using a combination of home visits and group meetings visits successfully reduced child body mass index (BMI) z-scores, and reduced the risk of overweight by 57% at 13–24 months of life [15].

The evidence base of increased risks associated with maternal obesity and can be used to inform preconception and pregnancy care [13,14,15]. Policy makers should emphasize the importance of supporting women to reduce their BMI preconception and inter-pregnancy, to prevent the adverse outcomes associated with post-term birth, such as perinatal and infant mortality [13,14,15].

There is evidence that a number of prenatal factors, such as maternal diet (total energy intake, macronutrient, and micronutrient composition), maternal obesity, microbiome, and environmental factors, can affect epigenomic regulation through altered gene and protein expression, leading to a higher risk of the offspring acquiring metabolic-syndrome related diseases [16,17]. In this specific window, also, the placenta is a critical organ, in that it transports nutrients from mother to fetus, and it is the interface between mother and fetus [18,19]. Pre-pregnancy obesity, excessive gestational weight gain, and gestational diabetes mellitus, could reflect maternal “overnutrition” and have been associated with larger placental size [18,20]. In this way the placenta plays a key role in controlling fetal growth, in fact, smaller placental size has been observed in pregnancies with intra-uterine growth restriction [18,21].

Hivert et al. using dual energy X-ray absorptiometry, a state of the art method to assess body composition, very recently found that the association between first trimester gestational weight gain and childhood adiposity is stronger in children born to women who have obesity before entering pregnancy [22]. In the same article, Hivert et al. also demonstrated for the first time that more rapid second trimester gestational weight gain is associated with greater lean mass (kg), particularly in women with normal weight prior pregnancy [22]. This suggests the need to address excess weight before conception, and gestational weight gain in very early pregnancy, especially in women with obesity. Using a national representative U.S. cohort, Hinkle et al. reported an increased risk for delayed mental development at age two years among children born to mothers who were underweight or obese before pregnancy [23]. Subjects in this study also presented with attention deficit hyperactivity disorder, autism or pervasive development disorder, intellectual disability, stuttering, or other developmental delays. It is hypothesized that the inflammatory intrauterine environment associated with pre-pregnancy BMI interrupts the fetal central nervous system development, and makes it more susceptible to other environmental insults by disrupting the blood brain barrier, all potentially leading to the increased risk for disabilities [23,24,25].

On the other hand, Shapiro et al. [26] found an association between poorer maternal diet quality and higher neonatal adiposity. This association is primarily related to the effect of lower diet quality on the body fat compartment of neonate, and this result is confirmed when the infant body fat is measured using precise and accurate body composition techniques [27]. Aris et al. showed that paternal overweight status [95% CI: 9.6–11.6] had a large individual predicted probability of child overweight/obese [28]. In light of these findings, early-life and preconception intervention programs may be more effective in preventing overweight and obesity occurring later in life [28].

Another point to consider is the type of birth delivery. A systematic review and meta-analysis suggests that cesarean birth is associated with higher risk of overweight and obesity in offspring [29]. A prospective cohort study including 22.068 offspring showed that cesarean birth was associated with a 45% increase in risk of obesity after adjusting for major confounding factors (i.e., age at delivery, ethnicity, gestational diabetes, pre-eclampsia, pre-pregnancy BMI, pre-pregnancy smoking, breastfeeding duration) [30].

Several articles found an inverse association between birth weight and adult blood pressure, or an inverse association between birth weight and risk of hypertension, as well as between birth weight and risk of coronary heart disease [31,32]. In this pathway, genetic factors may be involved, for example, genes that determine vascular endothelial function may be related to both resistance in the feto-placental circulation and the individual’s later risk of cardiovascular diseases [30].

Epigenetic factors also play a role, where modifiable factors affecting the intrauterine environment could program an individual’s susceptibility to disease [33,34]. Epigenetic modifications may represent a mechanism through which exposure to an altered intra-uterine milieu or metabolic perturbation may influence gene expression and modulate the phenotype of the organism much later in life [33,34,35].

These findings could suggest that parents’ behavior and nutrition, maternal obesity, excessive gestational weight gain, and type of delivery may be targets for intervention to prevent child obesity.

## 3. Practice Two: Systematize Body Composition Measurement to Monitor Growth

At birth, girls have proportionally more adipose tissue than boys (14.9% for girls and 13.7% for boys) [36] and it persists throughout life. Birth weight, length, and head circumference are the minimum measurements that are needed to be recorded at birth. Birth weight is highly objective and an easily measurable index, but greater sophistication in the area of measurement would be highly desirable [37,38,39]. Body composition assessment during infancy is important, because it is a critical period for obesity risk development, thus, valid tools are needed to accurately, precisely, and quickly determine both fat and fat-free mass [27]. Fields et al. [40], reported normative data on body composition in term infants, from birth to six months of age. It is important to note that lower-fat oxidation during the period of fastest growth (initial 6 months of life), and in the presence of positive energy balance, results in fat deposition [41]. After this initial period, there is a constant level of fat intake during the first two years, and increasing intake and oxidation of protein and carbohydrate [41]. Thus, beyond the first six months, with fat intake remaining unchanged, the growth of lean body mass relative to body weight accelerates [41]. It is fundamental to emphasize that weight gain in the second and third trimesters of fetal life, and in early, mid, and late infancy, is independently and positively associated with childhood BMI [42]. The strongest effects present for growth in late infancy and for BMI as an outcome, that children with both a high and a low birth weight, followed by infant growth acceleration, tend to have a higher BMI during childhood, and are at increased risk of overweight in childhood and adulthood [43].

The early years are the time of immense change when fundamental behaviors, including those around eating, sleeping, and physical activity, are established [38]. Understanding the changes in body composition that occurs during the first years of age, and how they may be related, may help inform evidence-based practice during childhood [39].

## 4. Practice Three: Exclusive Breastfeeding for the Best Start in Life

Results of several studies found breastfeeding was associated with a moderate, but consistent, protective effect against pediatric obesity [44,45,46]. Research suggests that infant feeding may influence the development of non-communicable diseases in adulthood. Breastfeeding is associated with a decreased risk of obesity and diabetes, as well as blood pressure [45,46]. Breast milk has long-chain polyunsaturated fatty acids (LC-PUFA), and supplementation with these fatty acids is associated with a reduction in blood pressure among adult subjects with hypertension [45,46]. Breastfed infants present with higher Bifidobacteria counts, and a lower count of these bacteria has been observed in fecal samples of obese children [47], showing that breast milk could offer an early protection against obesity. Based on the results from studies performed in both high-income and low- or middle-income settings, a meta-analysis found that breast-feeding was associated with a 13% reduction in overweight/obesity [48]. Harder et al. [49] found that each additional month of breastfeeding resulted in 4% lower obesity prevalence at later ages.

Breastfeeding, compared with bottle-feeding, may promote maternal feeding styles that are less controlling and more responsive to infant cues of hunger and satiety, thereby allowing infants greater self-regulation of energy intake [45,48,49].

We concluded that the majority of the evidence shows breastfeeding is associated with a moderate but consistent protective effect against later obesity. Clearly, these findings should encourage the promotion, protection, and support of breastfeeding, and of ethical approaches to the marketing of breast milk substitutes such as infant formula, as to not undermine breastfeeding [50]. Although, Lefebvre and John, in a systematic review on the effect of breastfeeding on childhood obesity, concluded that it is difficult to prove the protective benefits of breastfeeding because of confounding variables. Nevertheless, because of other benefits for the mother and the child, breastfeeding should be encouraged [51].

## 5. Practice Four: Window to Introduce Complementary Feeding

Complementary feeding is defined by the World Health Organization (WHO) [52] as the transition from breast milk to the family diet, and should occur when a baby is developmentally ready, and when breast milk is no longer enough to fulfill the nutritional requirements of the child. The WHO recommends exclusive breastfeeding until six months of age, after which breastfeeding should continue, but appropriate complementary foods should be introduced in a timely fashion [52].

The transition period is a fundamental milestone for every child. Pearce and Langly-Evans, in a systematic review, found that high energy intakes and high intake of protein, particularly animal protein, during the complementary feeding period, is associated with an increase childhood BMI, especially in the second year of life [53]. It is also clear that an early introduction of solid food (≤4 months of age) may result in an increase in childhood BMI [54], while no significant relationship was observed between delaying introduction of complementary foods after six months of age, and being overweight or obese during childhood [55]. Also, we need to emphasize that the iron status of healthy infants could be altered by an earlier introduction of complementary foods, leading to alteration of infant iron stores [56]. Higher energy intake during complementary feeding was associated with higher BMI in childhood. Adherence to dietary guidelines during weaning was associated with higher lean mass, while high intakes of energy and protein, particularly dairy protein, could be associated with an increase in body fatness [53]. Huh and colleagues, reported that introducing solid food before four months was associated with almost 6-fold increase in the risk of obesity among three-year-old formula-fed infants, but not in breastfed infants [57].

In conclusion, high intakes of energy and protein, particularly dairy protein in infancy, could be associated with an increase in BMI and body fatness. Weaning is an important time for the introduction of foods; so, adherence to dietary guidelines during weaning is recommended.

## 6. Practice Five: Fruit and Vegetable Liking Begins Early

Despite recommendations throughout Europe to increase children’s consumption of fruit and vegetables, they remain below recommendations in several countries [7,8,9,10]. Thus, it is crucial to establish preferences of fruit and vegetables when infants are “learning to eat”. Why it is so important the focus on fruit and vegetables? They are important sources of a wide range of vital micronutrients, and increased consumption of these foods can reduce the risks of a number of chronic diseases, including cardiovascular diseases [13,34,52,54].

Faith and colleagues found that a child’s diet is mediated through maternal foods [58], particularly for fruit and vegetables [59,60]. Complementary foods must embrace all food categories with an emphasis on vegetables and fruits [59]. Daily variety, diversity in a meal, and repeated exposure up to eight times are efficient strategies to increase acceptance of foods not initially accepted. Promoting healthy foods as part of usual meals during complementary feeding is important, as eating habits learned in childhood are likely to continue through life. There is no need to add sugar or salt to foods and sugar sweetened beverages (juice drinks, soda), and juices should be avoided during complementary feeding and onwards [61,62]. According with Faith et al. fruit juice intake was positively correlated with adiposity gain. This is particularly true in low-income families [62].

Several strategies are suggested for promoting fruit and vegetable consumption: (a) introducing fruit and vegetables early in the weaning process, (b) introducing a variety of fruit and vegetables, (c) repeating the presentation of a given fruit and vegetables several times, (d) offering fruit and vegetables in an appropriate way, with a sweet, sour or savory taste, (e) applying responsive feeding practices [7,8,9,10,13,34,52,54,60,61].

## 7. Practice Six: Respect for the Child’s Appetite

Eating behaviors develop early in life, and are a result of interactions between genetic predisposition, natural food responses and preferences and, more importantly, environmental influences. Parents influence a child’s weight through interactions that shape the development of child eating behaviors. Maternal autonomy promoting serving practices is positively associated with child appetite regulation [58,63].

Parental use of food as a reward leads to children’s diminished ability to regulate intake, which then leads to increased emotional over eating [64,65]. This suggests the need to assist children in learning how self-regulate in the presence of food [64,65]. Together with parental use of food as a reward, it is mandatory to avoid coercive “clean your plate” feedings practices. It is fundamental to adapt portions of the food and offer foods to the child in response to their feeling of hunger, and not to use foods as a reward for good behavior [58,64,65].

Recommendations are increasing toward the prioritization of vegetables, and the absence of repeated exposure to vegetables, up to eight times early in life, may induce a low capacity to taste different flavors later in life, and consequently promote picky eating [61,65].

Poor self-regulation of eating in children increases the risk of childhood obesity. While most children appear to possess an inborn ability to self-regulate food intake in responses to energy content of foods consumed, some children show poor patterns of self-regulation [64,65].

## 8. Practice Seven: Limit Animal Protein Intake

A high-protein intake in early childhood may increase the levels of insulin-releasing amino acids, which may in turn stimulate insulin and insulin-like growth factor (IGF-1) secretion, which are all upstream of the mammalian target of rapamycin growth signaling network [66]. Given that insulin plays a central role in metabolic regulation, attenuation of the elevated insulin secretion trough optimized by early nutrition, such as lower protein intake, could be a good suggestion. Also, the use of unmodified cows’ milk as a drink, which provides very high protein intake, in the first year of life should be discouraged.

**Figure 1:**
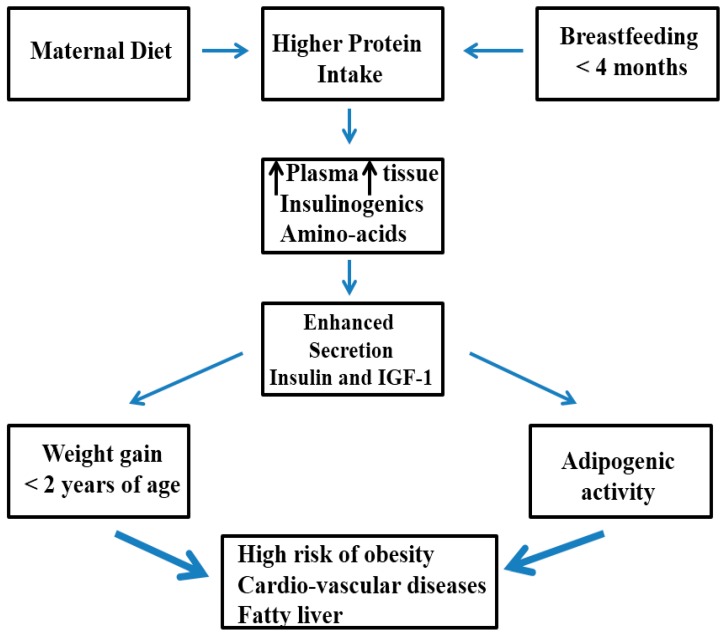
The early protein hypothesis suggests that a dietary protein supply in excess, together with early determinants, will lead to increased plasma and tissue concentration of insulin-releasing amino acids that ultimately results in adipogenic activity, and short- and long-term obesity risk.

The link to increased early protein intake with not only higher weight gain, but also obesity risk later in life, is that faster weight gain in infancy is associated with increased adipogenesis, and later, obesity risk [66]. Figure 1 shows the early protein hypothesis, and subsequently, the possible long-term obesity risk.

A large randomized controlled trial showed significant effects of a lower protein intake from infant formula on weight in the first two years of life [67]. Limiting the protein intake during infancy might constitute a potentially important approach to reducing the risk of childhood overweight and obesity [67]. Cow’s milk has a higher protein level compared to infant formula, and has a three times higher content of protein than human milk [66]. On the other hand, variation in the form of the protein content in formula fed to infants may relate to variations in cognitive development scores during the early infancy period [68].

Nutrient imbalance is particularly apparent in early childhood, with low fat and high protein diet. This is related to the fact that subjects need high energy for growth, and because it is the period of high rate of myelinization of the nervous system. At later ages, the proportion of fat exceeds the recommended level, and the protein intake remains high. A diet containing less animal and more vegetable products would reduce both protein and saturated fat intake, and could help decrease metabolic risk factors [69]. A higher intake of protein, especially at one year of age, was associated with a greater height, weight, and BMI in childhood up to nine years of age [69]. To meet the child nutritional requirements after 12 months, growing-up milk, named also young-child formulae [70], should be preferred to cow milk, in order to limit protein intake and meet essential fatty acid and iron needs [66].

## 9. Practice Eight: Promote Qualitative and Adequate Fat Intake

Early life stages can be seen as critical periods for fat cell development and adipose tissue growth in humans [71]. Immediately after birth, body fat accounts for approximately 14% of total body mass, and increases up to 20% at the age of one year. This increase in fat mass is mainly a result of an enlargement of existing fat cell size [72]. There is an association between early infant adipose deposition and overweight status in adults [73]. Infant exposure to high fatty acid intake as a mirror of maternal dietary fatty acid, may contribute significantly to adipose tissue deposition [74]. However, it was shown that infants that were exclusively or predominantly breastfed grew more rapidly in weight and length during the first months, and long-chain PUFAs (LC-PUFA) present in breast milk showed health benefits and positive effects against fat deposition [71]. Animal studies using n-3 LC-PUFA for anti-obesity effects showed decrease cellularity of adipose tissue and reduced lipid synthesis, which suggests a role for n-3 fatty acid in reducing both hyperplasia, as well as hypertrophy of growing fat depots [71].

Omega-3 and omega-6 fatty acids play an essential role in the development of the brain and the retina, and both of these fatty acids are essential for optimal development of brain and eyes [74]. A clinical deficiency of these fatty acids results in neurological abnormalities and poor growth, as fatty acids continue to accumulate most rapidly in the brain during the first two years of life.

Thus, the intake of adequate fat-containing essential fatty acids should be promoted. Low fat products should be avoided from weaning period and onwards [75,76], because food that is lower in fat may contain more sugar [77]. 

## 10. Practice Nine: Parents Be a Role Model

Dietary behaviors develop in the early years of life. During the second year of life, children share their food environment with parents and siblings in family. This shared “family food environment” influences children’s dietary intake, and provides a fundamental target setting for improving eating behaviors among children [59]. In relation to eating location, with eating while setting at a table, studies reported that it is associated with younger children’s increased fruit and vegetable consumption, appropriate portion size, social engagement between parents and children [58,60], and reduced access to TV viewing during meals. Children sometimes eat in the car or while playing and moving around the house. Screen time has been negatively associated with the development of physical and cognitive abilities, and positively associated with obesity, sleep problems, depression, and anxiety, even at an early age [78].

In addition to location, the context of eating is a fundamental part of eating time for children. The frequency of children watching TV while eating meals or snacks is related to consuming more unhealthy food and lower fruit and vegetables intake [58,59,60].

The American Academy of Pediatrics [79] recommend that parents limit children’s TV viewing, including computer/video/handheld game playing, to less than two hours per day. Watching TV more than two hours per day is associated with adverse health outcomes [80]. Moreover, young children are frequently exposed to advertisements for high-fat, high-sugar foods, which are linked to greater demand for and consumption of those foods. Restricting advertisements for these types of foods may be a viable obesity prevention strategy [80].

Parents need to be a role model for feeding. Parents behaviors should be a mirror for a child, making meals a pleasant experience spent with the family, starting with breakfast, respecting the child’s appetite and child specific nutritional needs with TV and all screens turned off [77].

## 11. Practice Ten: Promoting Physical Activity and Good Sleep

Promoting physical activity is a key component of preventing and controlling childhood obesity, even in subjects <5 years old [81]. Helping parents and their children develop the foundation for physical activity habits early in life is critical for the promotion of health in childhood and prevention of chronic diseases later in life, and ultimately will promote longer and healthier lives for individuals [82]. Saunders et al. [82] found that young children, with a combination of high physical activity and low sedentary behavior, had favorable measures of adiposity and cardio-metabolic health, when compared with those with a combination of low physical activity and high sedentary behavior. The available evidence suggests that optimal health benefits, even in early life, may come from replacing sedentary behavior with physical activity [83,84]. These findings suggest that there may be a synergistic benefit to achieving optimal levels of movement behaviors [82,83,84].

The term “lack of sleep” generally refers to an insufficient amount of sleep for optimal functioning [85]. The National Sleep Foundation recommends 10.5 to 18 h of sleep for subjects 1–2 months of life, 12 to 14 h for subjects 3 to 11 months and 12 h for subjects 1 to 3 years [85]. Sleep deprivation is a contributor to weight gain and subsequent obesity later in life [86,87]. Thus, it is fundamental to include sleep hygiene in health assessments and lifestyle modification interventions. Assessing general sleep hygiene (duration, quality, timing) does not need to be long and complicated, and can efficiently be incorporated into any health and lifestyle assessment. In this context, the American Academy of Pediatrics [79,80] recommends that parents remove TV and internet-connected electronic devices from children’s bedrooms.

Petrov and colleagues found that short sleep duration, <11 h, in subjects aged two to four years, was associated with greater fat and decreased carbohydrate consumption [86]. Fisher and colleagues provided the first evidence that shorter sleep is associated with higher energy intake in early childhood [87]. It is not possible to tell whether the increased energy intake was a physiological effect of shorter sleep, or the results of children being awake longer and having more time to eat [87]. However, we can conclude that there is a behavioral adaptation to short sleep in early life [85,86,87]. Small sustained changes over time have the potential to shift the population distribution of overweight and obesity [79].

In light of the above considerations, we can conclude that shorter night time sleep duration is associated with higher energy intake in early childhood before differences in weight have emerged. A higher energy intake is a possible mechanism through which shorter sleep contributes to adiposity in early life [87].

## 12. Conclusions

The obesity epidemic is an image of complexity, displaying a risk profile with biological and social susceptibilities across population groups, environments, and life courses that could be present even in early life [1,8,9,10]. The concept of early metabolic programming gave us the possibility to look at the literature, in order to see what causes could influence the first 1000 days of life, which is the most important and critical period for development. Different causes and effects are presented (see Table 1 for summary). The ten good practices developed have been shown to influence growth and function of different tissues in the human body. We found that excessive adipose tissue expansion may promote infant adipogenesis and infant rapid growth, both early markers of risk associated with childhood obesity.

Table 2 summarized and quantified the association between five peculiar interrelated behaviors, and overweight and obesity. We decided to look at parental overweight status before pregnancy, cesarean delivery, early infant feeding, short sleep duration, and physical activity, because these could be the five effective behaviors that play a role in pediatric overweight and obesity development [28,88,89,90,91].

Although, we need to underline that the article has two major limitations. First, our article is not a systematic review nor a meta-analysis of published data. Second, our paper is based on literature review, that a group of eminent researchers, experts in the field of pediatric obesity, derived from published science.

We could conclude that we need to know whether there are optimum times during the life span when experience promotes healthy food consumption for a healthy life, and conversely, when deprivation of such foods has the greatest consequences on health for generations to come [2,60,79]. The ten good practices together could help to prevent pediatric obesity and subsequent risks in adult life. These suggestions are not universally applicable, and are not easily used in low- or middle-income countries. Although, these ten good practices should be implemented and applied for a strong international code of food nutrition/market to monitor child growth in low- or middle-income countries, where in some cases, there is a more rapid increase in prevalence of overweight and obesity.

## Figures and Tables

**Table 1:** Summary of ten good practices to help prevent/combat pediatric obesity.

Practice	List	Reference
Practice 1	Both mother and father nutritional behavior matter	[6,12,13,14,15,16,17,18,19,20,21,22,23,24,25,26,27,28,29,30,31,32,33]
Practice 2	Systematize body composition measurements to monitor growth	[34,35,36,37,38,39,40,41]
Practice 3	Exclusive breastfeeding for the best start in life	[42,43,44,45,46,47,48]
Practice 4	Window to introduce complementary feeding	[49,50,52,53,54,55]
Practice 5	Fruits and vegetables liking begins early	[7,8,9,12,32,49,52,56,57,58,59,60]
Practice 6	Respect the child appetite	[56,61,62,63]
Practice 7	Limit animal protein intake	[64,65,66,67]
Practice 8	Promote qualitative and adequate fat intake	[69,70,71,72,73,74]
Practice 9	Parents be a role model	[56,57,58,77,78,79]
Practice 10	Promoting physical activity and good sleep	[80,81,82,83,84,85]

**Table 2:** Association between interrelate behaviors and overweight and obesity in childhood.

Behaviors Interrelate	Overweight and Obesity Risk	References
Parental overweight status		
Maternal	95% CI: 9.8–13.8	
Paternal	95% CI: 9.6–11.6	[28]
Cesarean delivery	95% CI: 1.10–4.27 (OR = 2.17)	[88]
Early infant feeding		
Longer breastfeeding—1 year	Beta = −0.027, *p* < 0.001	
3 years	95% CI: 1.43–2.94, *p* < 0.001	[89]
20 years	95% CI: 1.12–1.93, *p* = 0.005	
Short sleep duration in infancy	Beta = 0.16, 95% CI: 0.02–0.29	[90]
Physical activity		
Sedentary time	Beta = 0.042, 95% CI: −0.037 to 0.12	[91]
